# Engaging rural schools: Unique perspectives on stock inhaler policy implementation

**DOI:** 10.1016/j.anai.2025.08.729

**Published:** 2025-09-06

**Authors:** Emily Wilt, Ana Ongtengco, Paige Hardy, Molly A. Martin, Andrea A. Pappalardo

**Affiliations:** a University of Illinois Chicago College of Medicine, Chicago, Illinois; b Department of Pediatrics, University of Illinois Chicago, Chicago, Illinois; c Department of Medicine, University of Illinois Chicago, Chicago, Illinois

Asthma is the leading cause of health-related school absences nationwide.^1^ School-based asthma programs improve self-management and reduce caregiver anxiety.^2^ However, schools are often under-resourced and overburdened, preventing proactive management.^2^

To address asthma emergency preparedness needs at schools, Illinois passed Public Act 100–0726 in 2018, allowing trained school personnel to stock and administer undesignated rescue asthma medications, specifically albuterol inhalers. Stock inhaler programming improves medication access to asthma inhalers, improves school attendance, and reduces 9–1-1 calls to schools for asthma.^3^ The Illinois Department of Human Services provided $2.4 million to the RESCUE-IL (Resources for Every School Confronting Unexpected Emergencies-Illinois) program to provide eligible schools with stock inhalers and supplies. Eligible schools included publicly funded K-12 schools in Illinois based on enrollment and county-wide asthma risk, which was defined by pediatric asthma-related county-wide hospitalization and emergency department visits. Stock inhaler programming is slow to penetrate in rural schools, and 82.3% of ineligible RESCUE-IL schools (2023–2024 SY) were rural.^4,5^ Although the need for stock inhalers has been well documented, there has not been significant research done to investigate why rural schools specifically are not implementing stock inhaler programming. Therefore, a qualitative evaluation to identify barriers and facilitators to stock inhaler policy implementation in rural schools was completed to discover strategies that promote effective communication with rural schools.

Approval from the University of Illinois Chicago Institutional Review Board was obtained. Representatives from organizations with an interest in asthma, school health, and/or rural health were identified and contacted. Purposeful snowball sampling occurred. Recruitment was conducted through email. Demographic information was collected from the participants. The largest demographic represented was rural school health staff (n=8). Other participants included school administrators, clinicians, researchers, and asthma advocates. Virtual audio-only recorded interviews lasted 30 to 60 minutes. Recordings were transcribed and uploaded to Dedoose for analysis.

An interview guide was created *a priori*. Part 1 aimed to elucidate the participants’ role in rural school health. Part 2 encompassed school-based health initiatives, including questions on rural school engagement and previously successful communication strategies. Part 3 included questions surrounding participants’ knowledge of stock inhaler programming and perceived unique rural school benefits. Part 4 asked questions about collaboration with rural school health initiative implementation. Interviews were conducted until data saturation was achieved.^6^ A codebook was created *a priori*, and emergent codes were added as needed.^7^ Codes were organized into overarching themes through thematic analysis.^7^

Partner mapping revealed potential participants across a variety of interest levels and asthma policy influence. The 38 codes in the final codebook were distributed into 3 themes and 8 subthemes (Table 1).

The most significant theme, “barriers to stock inhaler implementation” included the subtheme “prominent rural school barriers.” The most frequently cited code in this subtheme, “lack of healthcare workers in the school,” cited in eight interviews, revealed that rural schools often lacked a school nurse or shared one nurse among multiple schools. Without a healthcare worker or designated school nurse or other trained school health personnel present, there is no one at the school trained in recognizing the symptoms of respiratory distress. In addition, although other staff, if trained, can administer stock inhalers under the current legislation, most do not feel comfortable administering medication without healthcare training, such as a nursing degree. Therefore, even if stock inhalers were brought into the school, their utilization would likely be lower. Outside of the school setting, many participants mentioned a “lack of access to primary care physicians” (9/21). Without primary care physicians, children who are experiencing trouble breathing are not diagnosed with asthma or treated. Those who have their first episode in school have difficulty finding a clinician to follow up with to ensure continued monitoring of their symptoms. With this lack of clinicians available in rural areas, long-term asthma control will not be addressed, even with the implementation of stock inhaler programming.

The second subtheme “common school barriers across geography” detailed barriers to stock inhaler implementation that were not unique to the rural setting. “Financial concerns,” cited in 11 interviews, explained the difficulties experienced when schools with limited budgets had to purchase stock inhalers and single-use chambers, which often are costly. Other barriers included “denying asthma need” (6/21), revealing how often schools viewed asthma as less of a priority, making them less likely to invest in school-based asthma care.

Other themes discovered through this research can be found in Table 1 and include “rural school engagement,” which houses 13 codes that detail how participants had previously engaged with rural schools on other health-related programming. This theme houses the following three subthemes: “barriers to engagement,” “facilitators to engagement,” and “successful communication methods.” The final theme, “stock inhaler program necessity” details why stock inhalers are needed in schools, particularly in the rural setting. This code is broken down into the following two subthemes: “school-based factors” and “community/external factors,” detailing reasons both within the school setting and outside of it that make access to stock inhaler medications necessary.

This qualitative study of rural school health partners emphasized the difficulties schools encounter in accessing quality health care and the unique need for stock inhaler programming. School-based health programming is especially important for children with asthma in rural areas due to well-documented limitations in health care access. As of 2020, only 11% of physicians practiced in rural areas, and rural residents were 10% to 20% less likely to receive preventive medical care.^8^ Greater distance to health care, frequently cited in our study, is also a risk factor for morbidity and mortality and poorer health outcomes in patients with asthma.^9^ These factors leave children with asthma vulnerable to experiencing symptoms without the infrastructure to recognize, manage, and refer them for further services.

Stock inhaler programming implementation can improve emergency response, but additional school health programming and improvement of communication between schools, communities, and healthcare systems are also necessary. Only 33% of children with asthma seek medical attention for their symptoms, demonstrating a concern regarding asthma management resources in rural communities outside of the school setting.^10^ Many children in rural areas are uninsured, under-insured, or on public insurance. Therefore, having access to inhalers at school is especially important to address the lack of external access to medications.^10^

This study demonstrated that clear communication, engaging a trusted community champion, and securing funding are essential to increasing stock inhaler programming in rural schools. Limitations to this study include a small sample size, which limits generalizability and external validity and may introduce bias. Future research could explore partnerships with influential organizations in the field of asthma care, such as the American Lung Association and Breathmobile programs. Rural schools want and need stock inhalers but have significant barriers that must be addressed for programming to be successful, most notably financial strains and a lack of health care workers in the school. Effective partnerships and communication strategies drive solutions toward comprehensive, guideline-concordant school-based asthma management.

## Figures and Tables

**Table 1:** Rural School Partner Key Informant Interview Thematic Analysis Summary

Code	No. of interviews code was cited in (n = 21)	Subthemes	Themes

School competing priority related to health	10	Barriers	Rural school engagement
Distrust of outside authority	6		
Schools overwhelmed	5		
Lack ofhealth care access	3		
Concern of liability for misuse	3		
School competing priority not related to health	2		
Administration on board	7	Facilitators	
Community involvement	6		
External support	6		
Community support	14	Successfully used communication methods	
Clear communication	11		
Personalized communication	4		
Legal support	3		
De-escalation of symptoms	12	Medical factors in schools	Stock inhaler program necessity
High need for inhalers	5		
Secured a provider to write a prescription for albuterol	3		
Facilitates follow-up	6	Non-medical factors in schools	
Peace of mind	6		
Facilitates asthma education	5		
Secured funding	3		
Reassurance	2		
Lack of personal inhalers	12	Medical community/external factors	
Inhaler accessibility	10		
High asthma prevalence	9		
Environmental exposures/asthma triggers	6		
Underdiagnosis of asthma	5		
Distance to emergency services	15	Non-medical community/external factors	
Lack of external resources	8		
Lack of access to primary care physician	9	Prominent rural school barriers	Barriers to stock inhaler implementation
No health care workers in the school	8		
Uncomfortable with stock inhaler use	5		
Unaware of stock inhaler program	2		
Financial concerns	11	Common school barriers across geography	
Denying need	6		
Sufficient inhaler supply	6		
Uncertain of protocols	5		
Stock inhaler medication/prescription issues	4		
Charting/documentation	2		
